# Characterization and phylogenetic analysis of the mitochondrial genome of *Holotrichia parallela* (Coleoptera: Scarabaeidae: Melolonthinae)

**DOI:** 10.1080/23802359.2021.2016082

**Published:** 2022-01-18

**Authors:** Jing Zhao, Feng Zhang, Jingjing Yuan, Zhenjie Du, Xuebin Qi

**Affiliations:** a College of Materials Science and Engineering, Henan Institute of Technology, Xinxiang, China; b Farmland Irrigation Research Institute, Chinese Academy of Agricultural Sciences, Xinxiang, China; c Key Laboratory of High-efficient and Safe Utilization of Agriculture Water Resources of CAAS, Xinxiang, China; d College of Intelligent Engineering, Henan Institute of Technology, Xinxiang, China; e Henan Institute of Metrology, China Henan Institute of Metrology, Xinxiang, China

**Keywords:** Scarabaeidae, *Holotrichia parallela*, mitochondrial genome, phylogenetic analysis

## Abstract

*Holotrichia parallela* (Motschulsky, 1854) is an important pest for peanut, potato, and soybean in China, and it causes great economic losses. In this study, we sequenced and analyzed the complete mitochondrial genome (mitogenome) of *H. parallela*. This mitogenome was 16,975 bp long and encoded 13 protein-coding genes (PCGs), 22 transfer RNA genes (tRNAs), and two ribosomal RNA genes (rRNAs). Gene order was conserved and identical to most other previously sequenced Scarabaeidae. Most PCGs of *H. parallela* have the conventional start codons ATN, with the exception of *cox1* (AAC). Except for three genes (*cox1*, *cox2*, and *cox3*) end with the incomplete stop codon T−, all other PCGs terminated with the stop codon TAA or TAG. Phylogenetic analysis positioned *H. parallela* in a well-supported clade with *Rhopaea magnicornis*, *Polyphylla gracilicornis*, and *Melolontha hippocastani*. The relationships (Dynastinae+(Cetoniinae+(Melolonthinae+(Rutelinae + Scarabaeinae)))) were supported in Scarabaeidae.

The Scarabaeidae is the largest of the 13 families in the Scarabaeoidea and one of the largest and most diverse family of beetles. They are often called scarabs or scarab beetles and consist of over 30,000 species worldwide (Browne and Scholtz 2008). The morphological and biological diversity of its members has led to the family being divided into numerous mostly well-defined subfamilies and tribes, as well as into several groups of uncertain taxonomic status (Browne and Scholtz 1999). One of its species, the large black chafer *Holotrichia parallela* (Motschulsky, 1854), is a world-wide pest of many crops, which can cause serious damage to peanut, potato, soybean, and turf grasses, resulting in 15–20% losses annually (Ju et al. 2012). Mitogenome can be utilized in research on population genetic structure, taxonomic resolution, phylogeography, and phylogeny. For further study on population genetic structure of *H. parallela*, we sequenced the complete mitogenome of *H. parallela* and analyzed the phylogenetic relationships of Scarabaeidae based on mitogenome data.

Male adults of *H. parallela* were collected from a pig farm of Nanyang City, Henan Province, China (33°06′N, 111°36′E, July 2019) and were stored in Entomological Museum of Henan Institute of Technology (accession number QHU-EHP03, Dr. Jing Zhao, 64282381@163.com). Total genomic DNA was extracted from muscle tissues of the thorax using DNeasy DNA Extraction kit (Qiagen, Hilden, Germany). A pair-end sequence library was constructed and sequenced using Illumina HiSeq 2500 platform (Illumina, San Diego, CA), with 150 bp pair-end sequencing method. A total of 24.6 million reads were generated and had been deposited in the NCBI Sequence Read Archive (SRA) with accession number SRR14149021. Raw reads were assembled using MITObim v 1.7 (Hahn et al. 2013). By comparison with the homologous sequences of other Scarabaeidae species from GenBank, the mitogenome of *H. parallela* was annotated using software GENEIOUS R11 (Biomatters Ltd., Auckland, New Zealand).

The complete mitogenome of *H. parallela* is 16,975 bp in length (GenBank accession no. MW874410), and contains the typical set of 13 protein-coding, two ribosomal RNA (rRNA) and 22 transfer RNA (tRNA) genes, and one non-coding AT-rich region. Gene order was conserved and identical to most other previously sequenced Scarabaeidae (Cameron et al. 2009; Shao et al. 2014; Jing et al. 2018; Jeong et al. 2020; Yang et al. 2020). The nucleotide composition of the mitogenome is 75.2% A + T content (A 40.2%, T 35.0%, C 16.2%, and G 8.6%). Four protein-coding genes (PCGs) (*nad4*, *nad4l*, *nad5*, and *nad1*) were encoded by the minority strand (N-strand) while the other nine were located on the majority strand (J-strand). All PCGs of *H. parallela* have the conventional start codon for invertebrate mitochondrial PCGs (ATN), with the exception of *cox1* (AAC), as the asparagine (AAT or AAC) are proposed to be the start codon for *cox1* in suborder Polyphaga (Sheffield et al. 2008). Except for three genes (*cox1*, *cox2*, and *cox3*) end with the incomplete stop codon T−, all other PCGs terminated with the stop codon TAA or TAG. The 22 tRNA genes vary from 62 bp (*trnC* and *trnD*) to 70 bp (*trnQ*, *trnK*, and *trnV*). Two rRNA genes (*rrnL* and *rrnS*) locate at *trnL1*/*trnV* and *trnV*/control region, respectively. The lengths of *rrnL* and *rrnS* in *H. parallela* are 1288 and 755 bp respectively, with AT contents of 78.1% and 72.4%, respectively.

Phylogenetic analysis was performed based on the nucleotide sequences of 13 PCGs from 22 Polyphaga species. Alignments of individual genes were concatenated using SequenceMatrix 1.7.8 (Vaidya et al. 2011). Phylogenetic tree was constructed through raxmlGUI 1.5 (Silvestro and Michalak 2012). Phylogenetic analysis positioned *H. parallela* in a well-supported clade with *Rhopaea magnicornis*, *Polyphylla gracilicornis* and *Melolontha hippocastani* (Figure 1), indicating genus *Holotrichia* had a close relationship with *Rhopaea*, *Polyphylla*, and *Melolontha* within Melolonthinae. The relationships (Dynastinae+(Cetoniinae+(Melolonthinae+(Rutelinae + Scarabaeinae)))) were supported in Scarabaeidae. The monophyly of Melolonthinae could not be confirmed by this phylogenetic tree. These results provided an important basis for further studies on mitochondrial genome and phylogenetics of Scarabaeidae.

**Figure 1.**
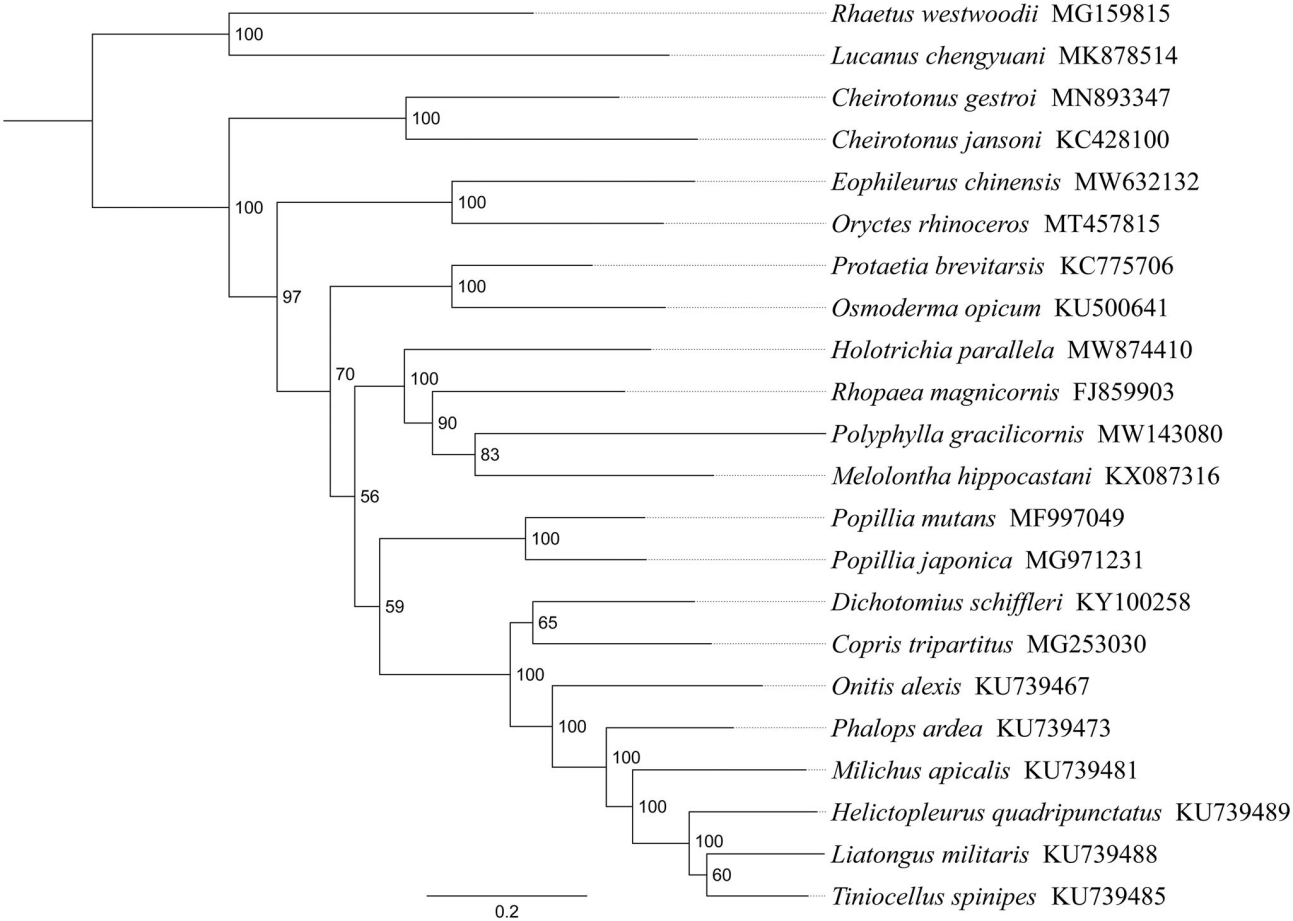
Phylogenetic relationships based on the 13 mitochondrial protein-coding genes sequences inferred from RaxML. Numbers on branches are bootstrap support values (BS).

## Data Availability

The data that support the findings of this study are openly available in NCBI (National Center for Biotechnology Information) at https://www.ncbi.nlm.nih.gov/, reference number MW874410. The associated BioProject, SRA, and Bio-Sample numbers are PRJNA719935, SRR14149021, and SAMN18628722, respectively.
